# Serum Vitamin D Levels and Polycystic Ovary syndrome: A Systematic Review and Meta-Analysis

**DOI:** 10.3390/nu7064555

**Published:** 2015-06-08

**Authors:** Chunla He, Zhoumeng Lin, Sara Wagner Robb, Amara E. Ezeamama

**Affiliations:** 1 Department of Epidemiology and Biostatistics, College of Public Health, University of Georgia, 101 Buck Road, Health Sciences Campus, B.S. Miller Hall, Athens, GA 30602, USA; E-Mails: willahe@uga.edu (C.H.); swagner@uga.edu (S.W.R.); 2 Institute of Computational Comparative Medicine (ICCM), Department of Anatomy and Physiology, College of Veterinary Medicine, Kansas State University, Manhattan, KS 66506, USA; E-Mail: zhoumeng@ksu.edu

**Keywords:** vitamin D, polycystic ovary syndrome, metabolic and endocrine disorders, systematic review and meta-analysis

## Abstract

Vitamin D deficiency (VDD) is common in women with and without polycystic ovary syndrome (PCOS) and may be associated with metabolic and endocrine disorders in PCOS. The aim of this meta-analysis is to assess the associations of serum vitamin D levels with metabolic and endocrine dysregulations in women with PCOS, and to determine effects of vitamin D supplementation on metabolic and hormonal functions in PCOS patients. The literature search was undertaken through five databases until 16 January 2015 for both observational and experimental studies concerning relationships between vitamin D and PCOS. A total of 366 citations were identified, of which 30 were selected (*n* = 3182). We found that lower serum vitamin D levels were related to metabolic and hormonal disorders in women with PCOS. Specifically, PCOS patients with VDD were more likely to have dysglycemia (e.g., increased levels of fasting glucose and homeostatic model assessment-insulin resistance index (HOMA-IR)) compared to those without VDD. This meta-analysis found no evidence that vitamin D supplementation reduced or mitigated metabolic and hormonal dysregulations in PCOS. VDD may be a comorbid manifestation of PCOS or a minor pathway in PCOS associated metabolic and hormonal dysregulation. Future prospective observational studies and randomized controlled trials with repeated VDD assessment and better characterization of PCOS disease severity at enrollment are needed to clarify whether VDD is a co-determinant of hormonal and metabolic dysregulations in PCOS, represents a consequence of hormonal and metabolic dysregulations in PCOS or both.

## 1. Introduction

Polycystic ovary syndrome (PCOS) [1] is the most common female endocrine disorder, affecting approximately 4%–18% women of reproductive age [2,3,4,5]. It is a heterogeneous androgen excess disorder with different degrees of reproductive and metabolic dysfunctions. Metabolic disturbances including insulin resistance, hyperinsulinemia and dyslipidemia, are common features in the majority of women with PCOS [6,7,8,9,10,11,12]. Women with PCOS may also be at elevated risk of vitamin D deficiency (VDD). In contrast to a prevalence of 20%–48% among the general adult population [13,14,15], a relative higher prevalence of VDD is observed among women with PCOS (approximately 67%–85% women with PCOS have VDD [16]). Additionally, positive associations of VDD with some well-known comorbiditiesof PCOS including type 2 diabetes, insulin resistance, metabolic syndrome, and cardiovascular diseases, are reported [17,18,19,20]. In this regard, an increasing number of studies have been conducted to investigate the specific relationship between vitamin D status and PCOS. Although several studies have suggested that lower vitamin D levels are associated with increased risk of insulin resistance and metabolic disturbance among women with PCOS [21,22], the current findings are inconsistent.

Vitamin D receptors are expressed in 2776 genomic positions and modulate the expression of 229 genes in more than 30 different tissues, such as skeleton, brain, breast, pancreas, parathyroid glands, immune cells, cardiomyocytes, and ovaries [23,24]. Thus, deficiency in this vitamin, in addition to its well-described role in calcium homeostasis and bone metabolism, may cause a wide range of extra-skeletal effects with impact on glucose homeostasis, cardiovascular disease, cancer, autoimmune diseases and psychological disorders [23,25,26,27]. Vitamin D may play a role in glucose metabolism by enhancing insulin synthesis and release, and increasing insulin receptor expression or suppression of proinflammatory cytokines that possibly contribute to the development of insulin resistance [28]. The effect of vitamin D on metabolic and reproductive dysfunctions in PCOS may be mediated by insulin resistance. Reproductively, insulin resistance increases hyperandrogenism through insulin increasing ovarian androgen production, and reducing sex hormone-binding globulin (SHBG) production [29]. Metabolically, insulin resistance is associated with an increased risk for impaired glucose tolerance, type 2 diabetes mellitus and cardiovascular disease [30,31,32,33,34]. Therefore, vitamin D may play a key role in the development of PCOS.

Associations of vitamin D status with PCOS, metabolic and hormonal dysfunctions in PCOS in particular, have been investigated by a large number of studies, but the relationship between them remains inconclusive. In spite of a growing number of intervention studies assessing effects of vitamin D supplementation on PCOS, a lack of convincing evidence demonstrating a causal link between low vitamin D levels and PCOS exists, mainly due to small sample sizes (the majority of them has a sample size less than 30). Therefore, the objective of this systematic review and meta-analysis is to quantitatively summarize existing evidence to determine whether serum vitamin D concentrations are lower in women with PCOS compared to women without PCOS, to determine whether vitamin D deficiency is associated metabolic and endocrine dysregulations in women with PCOS, and to evaluate effects of vitamin D supplementation on the mitigation of metabolic and hormonal functions in women with PCOS.

## 2. Materials and Methods

### 2.1. Search Strategy

Relevant studies were identified from the following electronic databases: PubMed, Web of Science, Cochrane Central Register of Controlled Trials (CENTRAL), Cumulative Index to Nursing and Allied Health (CINAHL), and PsycINFO. Databases were searched using the search strategy as shown in Supplementary Table S1, from the earliest available date to 16 January 2015. We also manually searched reference lists of all eligible articles and previous reviews on relevant topics for additional studies.

### 2.2. Study Selection 

Studies were included in the review if they fulfilled the following criteria: (1) published in the English language; (2) included women with PCOS; (3) presented (3.1) comparison of serum vitamin D concentrations between women with and without PCOS; (3.2) comparison of metabolic or endocrine parameters between VDD and non-VDD groups, between vitamin D intervention and placebo groups, or between pre- and post-intervention of vitamin D supplementation; or (3.3) correlation between 25(OH)D and metabolic or endocrine indices in women with or without PCOS.

Studies were excluded from this meta-analysis if they were publications from meetings/congress or were genetic studies that did not provide baseline descriptions of metabolic or endocrine parameters. For studies with more than one article based on the same study population, inclusion was limited to the one with the most recent publication date or with the largest sample size.

### 2.3. Data Extraction

Two reviewers (C.H. and Z.L.) independently identified and selected articles that met the inclusion criteria. Discrepancies were resolved by consensus and arbitration (C.H., Z.L., and A.E.E.). General characteristics of the study (e.g., author, year of publication, study location), characteristics of the study population (e.g., recruitment source, sample size, mean age and body mass index (BMI)), definition of PCOS, measurement of variables of interest (e.g., serum vitamin D levels; metabolic parameters, such as fasting glucose, fasting insulin, homeostatic model assessment-insulin resistance index (HOMA-IR), homeostatic model assessment-β-cell functions (HOMA-β), quantitative insulin sensitivity check index (QUICKI), total cholesterol, triglycerides, high density lipoprotein cholesterol (HDL-C), low density lipoprotein cholesterol (LDL-C), C-reactive protein (CRP); and endocrine indicators including total testosterone, free testosterone, free androgen index (FAI), sex hormone-binding globulin (SHBG), and dehydroepiandrosterone sulfate (DHEAS)) were extracted from included studies.

### 2.4. Quality Assessment

The quality of studies was examined and controlled in accordance with checklists of Preferred Reporting Items for Systematic reviews and Meta-Analyses for randomized trials [35], and checklists of Meta-analysis of Observational Studies in Epidemiology (MOOSE) for observational studies [36]. No scoring methods were utilized for assessment of quality of studies due to a lack of reliable and standardized scales for observational studies [37,38,39].

### 2.5. Data Analysis

We calculate five types of effect sizes in this study: (1) standardized mean differences (SMDs) in serum vitamin D levels between PCOS and non-PCOS groups; (2) SMDs in metabolic and endocrine indices between VDD and non-VDD groups; (3) correlations between 25(OH)D and metabolic or endocrine parameters; (4) pre- *versus* post-intervention SMDs in vitamin D concentrations, metabolic and endocrine parameters; and (5) post-intervention SMDs in vitamin D levels, metabolic and endocrine parameters between vitamin D supplementation and placebo groups. The size of the SMD can be interpreted as being small (<0.2), medium (0.2–0.8), or large (>0.8) [40]. Correlation values of less than 0.1, of 0.1–0.5, of greater than 0.5, are considered to be indicative of small, medium and large effect size, respectively [40]. If a study presented analyses stratified by certain key variables such as BMI, stratified estimates were assumed to be independent of each other and included as a separate unit of observation in the meta-analysis. For example, Panidis *et al.* performed stratified comparisons of serum vitamin D concentrations according to BMI values (*i.e.*, obese PCOS *vs.* obese controls, overweight PCOS *vs.* overweight controls, normal weight PCOS *vs.* normal weight controls) [41], and thus three observations were obtained from this study. In order to adjust for bias resulting from small sample sizes, between-group SMDs were calculated using Hedges’ formula [42]. Papers which did not present the mean and standard deviation (SD), values of median (m) and range (a and b represent low and high end of range, respectively) were converted into mean and SD based on formulas as follows: $\bar{x}\approx \frac{a+2m+b}{4}$, $\;S^{2}\approx \frac{1}{12}\left(\frac{{\left(a-2m+b\right)}^{2}}{4}+{\left(b-a\right)}^{2}\right)\;$ [43], where $\bar{x}$ and $S^{2}$ refer to the values of mean and variance, respectively. Correlations between serum 25(OH)D levels and metabolic and hormonal parameters, were also summarized in this study. Because data for some variables in the original studies were log-transformed, Pearson correlation coefficients were converted into Spearman correlation coefficients according to the formula: ${\text{r}}_{\text{s}}\text{=}\frac{\text{6}}{\pi }sin^{-1}\left(\frac{r}{2}\right)$ , where $r_{s}\;$ and r  are Spearman and Pearson correlation coefficients, respectively [44]. The sampling distribution of Spearman correlation coefficients is problematic because its standard error (SE) depends on the value of the correlation coefficient. Thus, a Fisher transformation formula showed as follows was used to convert each $r_{s}$ into an approximately normally distributed variable  z with $SE=\frac{1}{\sqrt{n-3}}$ (n is the sample size): $z=\frac{1}{2}\left(\ln\left(1+r_{s}\right)-\ln\left(1-r_{s}\right)\right)$ . After appropriate conversion, the inverse variance-weighted method was used to estimate effect size and corresponding 95% confidence intervals (CIs). The Fisher-transformed data were converted back to the original scale for interpretation. Heterogeneity across studies was tested using Cochran’s Q and *I^2^* statistic [45]. A *p*-value less than 0.1 from the Q statistic was considered to be indicative of statistically significant heterogeneity. The *I^2^* statistic was calculated to express the fraction of variation between studies that was due to heterogeneity [45]. *I^2^* values of 25%, 50% and 75% were considered as low, moderate and high heterogeneity, respectively [45]. The pooled effect size was estimated based on the fixed effects model when no significant heterogeneity was detected. Otherwise, a random effects model was used [46]. Sensitivity analyses in which one study at a time was omitted from the effect size calculations were undertaken to determine whether the pooled effect size was unduly influenced by a specific study. Publication bias was examined using a funnel plot and Egger’s test [47]. All analyses were performed in STATA 12 (StataCorp, College Station, TX, USA). *p*-Values < 0.05 were considered statistically significant for all analyses except heterogeneity tests.

## 3. Results

### 3.1. Study Selection

**Figure 1:**
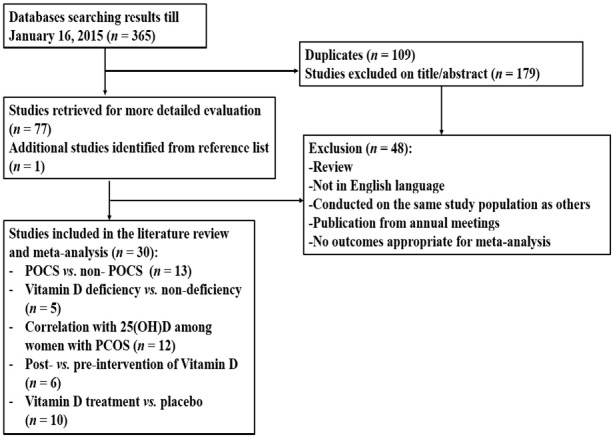
Flowchart of literature search.

The search yielded 365 citations as shown in Figure 1. Initial screening of the title and abstract resulted in the exclusion of 179 references and 77 studies proceeded to detailed evaluation. One additional reference [48] was identified by searching the reference lists of the 77 full text papers. After further examination, 30 studies met the inclusion criteria and were included in the meta-analysis.

### 3.2. Characteristics of Included Studies

A total of 30 studies involving 3182 participants were included in the meta-analysis. Characteristics of included studies are presented in Table 1. Twenty-five studies defined PCOS based on criteria of Rotterdam European Society for Human Reproduction & Embryology/American Society for Reproduction Medicine (ESHRE/ASRM) PCOS Consensus Workshop Group [1], while five studies diagnosed PCOS using the National Institutes of Health criterion [49]. Eleven studies were conducted in Europe, ten in Asia, three in U.S., three in Turkey, and three in Egypt. Thirteen studies compared 25(OH)D and/or 1,25(OH)_2_D levels between women with or without PCOS; five studies compared metabolic or hormonal parameters between VDD and non-VDD groups; twelve studies presented correlation coefficients between 25(OH)D and metabolic/endocrine indices among women with PCOS; ten studies compared biochemical outcomes between pre- and post-intervention of vitamin D supplementation; and six studies compared post-intervention differences in metabolic and endocrine parameters between vitamin D supplementation and placebo groups.

### 3.3. Differences in Vitamin D Levels between PCOS Patients and Controls

**Figure 2:**
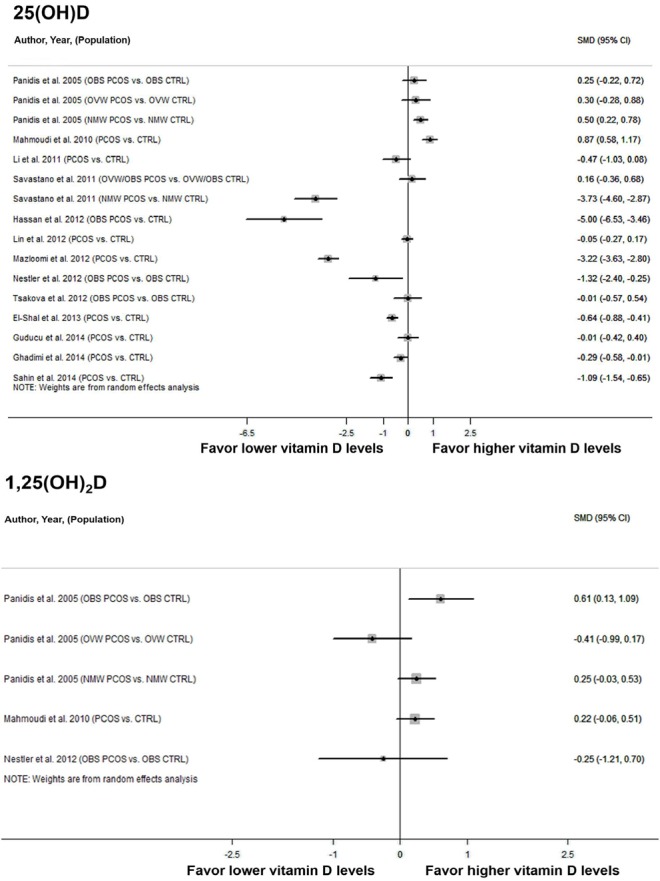
Forest plots showing the effect size of the association between serum vitamin D levels and PCOS in women (SMD, standardized mean difference; OBS, obese; OVW, overweight; NMW, normal weight; PCOS, polycystic ovary syndrome; CTRL, control.).

Among thirteen studies comparing 25(OH)D levels between women with and without PCOS, two presented stratified statistics in terms of BMI values [41,50], therefore yielding a total of 16 observations for calculation of effect size for 25(OH)D levels. A random effects model revealed a moderate estimate of effect size (SMD: −0.74, 95% CI: −1.26 to −0.22) (Figure 2, top), which indicated that serum 25(OH)D concentrations were significantly lower in PCOS patients compared to controls without PCOS. Significant heterogeneity was identified across included studies (*p* < 0.001, *I^2^* = 96.5%). Sensitivity analyses were conducted to assess the extent to which individual studies with extremely large SMDs influenced the pooled SMD. No substantially influential test was identified from the sensitivity analyses. Omitting two studies [48,51] with small sample size (<30) resulted in insignificant estimates of SMD (−0.50; 95%CI: −1.03 to 0.03). Egger’s test (*p* = 0.298) and visual examination of funnel plots indicated no significant publication bias over all included studies.

Three studies involving five observations were included in the estimate of effect size for 1,25(OH)_2_D. Heterogeneity between studies was statistically significant (*p* = 0.091, *I^2^* = 50.1%). No significant difference in 1,25(OH)_2_D was found between PCOS patients and controls (SMD: 0.18; 95%CI: −0.10 to 0.45) (Figure 2, bottom). The Funnel plot and Egger’s test (*p* = 0.453) suggested no significant publication bias over the included studies.

### 3.4. Comparison in Metabolic and Endocrine Indices in PCOS between VDD and Non-VDD Women

Being vitamin D deficient was significantly associated with lower HDL-C, and with higher fasting glucose, fasting insulin, HOMA-IR, HOMA-β, and FAI among women with PCOS (Table 2). No significant heterogeneity was found in studies reporting fasting glucose, fasting insulin, HOMA-β, HDL-C, LDL-C and FAI. No significant publication bias was identified from all reported parameters.

**Table 1:** Characteristics of included studies in the meta-analysis.

Author (Year)	Location	Diagnosis	Participants (*n*)	Variables
**Comparison between PCOS Patients and Control Women**				
Panidis *et al.*, (2005) [41]	Greece	ESHRE/ASRM	PCOS,(291); healthy CTRL,(109)	25(OH)D; 1,25(OH)2D
Mahmoudi *et al.*, (2010) [52]	Iran	NIH	PCOS, (85); CTRL, (115)	25(OH)D; 1,25(OH)2D
Li *et al.*, (2011) [53]	UK	ESHRE/ASRM	PCOS, (25); CTRL, (27)	25(OH)D
Savastano *et al.*, (2011) [50]	Italy	ESHRE/ASRM	PCOS, (90); Healthy CTRL, (40)	25(OH)D
Hassan *et al.*, (2012) [48]	Egypt	ESHRE/ASRM	PCOS, (30); CTRL, (15)	25(OH)D
Lin *et al.*, (2012) [54]	Taiwan	ESHRE/ASRM	PCOS, (188); CTRL, (143)	25(OH)D
Mazloomi *et al.*, (2012) [55]	Iran	ESHRE/ASRM	PCOS, (103); healthy CTRL, (103)	25(OH)D
Nestler *et al.*, (2012) [51]	USA	NIH	Obese PCOS, (8); Obese CTRL, (9)	25(OH)D; 1,25(OH)2D
Tsakova *et al.*, (2012) [56]	Bulgaria	ESHRE/ASRM	Obese PCOS, (20); Obese CTRL, (33)	25(OH)D
El-Shal *et al.*, (2013) [57]	Egypt	ESHRE/ASRM	PCOS, (150); CTRL, (150)	25(OH)D
Guducu *et al.*, (2014) [58]	Turkey	ESHRE/ASRM	PCOS, (58); CTRL, (38)	25(OH)D
Ghadimi *et al.* (2014) [59]	Iran	ESHRE/ASRM	PCOS, (104); CTRL, (88)	25(OH)D
Sahin *et al.* (2014) [60]	Turkey	ESHRE/ASRM	Lean PCOS, (50); CTRL (40)	25(OH)D
**Comparison between Vitamin D Deficient and Non-Deficient PCOS Patients**				
Wehr *et al.*, (2009) [22]	Austria	ESHRE/ASRM	PCOS, (206)	FG; HOMA-IR; HOMA-β; QUICKI; FI; TC; TG; HDL-C; LDL-C; CRP; TT; FT; SHBG; FAI
Li *et al.*, (2011) [53]	UK	ESHRE/ASRM	PCOS, (25)	FG; FI; HOMA-IR; HOMA-β; QUICKI; TC; HDL-C; LDL-C; TG; CRP; TT; SHBG; FAI
Patra *et al.*, (2012) [61]	India	ESHRE/ASRM	PCOS, (60)	HOMA-IR
Bhattacharya *et al.*, (2013) [62]	India	ESHRE/ASRM	PCOS, (93)	TT; SHBG; FAI; FG; FI
Velija-Asimi *et al.*, (2014) [63]	Bosnia and Herzegovina	ESHRE/ASRM	PCOS, (60)	TC; TG; CRP; FG; FI; TT; HOMA-IR; SHBG
**Correlation between 25(OH)D and Metabolic and Endocrine Parameters among Women with PCOS**				
Hahn *et al.*, (2006) [64]	Germany	NIH	PCOS, (120)	HOMA-IR; HOMA-β; QUICKI; TG; HDL-C; LDL-C; FG; TT;FAI; SHBG; DHEAS
Wehr *et al.*, (2009) [22]	Austria	ESHRE/ASRM	PCOS, (206)	FG; HOMA-IR; FI; TC; TG; HDL-C; LDL-C; CRP; TT; FT; SHBG; FAI
Yildizhan *et al.*, (2009) [65]	Turkey	ESHRE/ASRM	PCOS, (100)	HOMA-IR; TC; TG; TT; DHEAS;
Li *et al.*, (2011) [53]	UK	ESHRE/ASRM	PCOS, (25)	FG; FI; HOMA-IR; HOMA-β; QUICKI; TC; HDL-C; LDL-C; TG; CRP; TT; SHBG; FAI
Savastano *et al.*, (2011) [50]	Italy	ESHRE/ASRM	PCOS, (90)	HOMA-IR; FI; FAI
Bonakdaran *et al.*, (2012) [66]	Iran	ESHRE/ASRM	PCOS, (51)	DHEAS
Patra *et al.*, (2012) [61]	India	ESHRE/ASRM	PCOS, (60)	HOMA-IR; FG
El-Shal *et al.*, (2013) [57]	Egypt	ESHRE/ASRM	PCOS, (150)	TC; TG; HDL-C; LDL-C; FG; FI; HOMA-IR; HOMA-β; QUICKI; TT; FT; SHBG; DHEAS
Guducu *et al.*, (2014) [58]	Turkey	ESHRE/ASRM	PCOS, (58)	FI
Kozakowski *et al.* (2014) [67]	Poland	ESHRE/ASRM	Obese PCOS, (60)	TC; HDL-C; LDL-C; TG; FG; FI; TT; DHEAS; FAI; SHBG
Ghadimi *et al.* (2014) [59]	Iran	ESHRE/ASRM	PCOS, (104)	HOMA-IR
Sahin *et al.* (2014) [60]	Turkey	ESHRE/ASRM	Lean PCOS, (50)	HOMA-IR
**Comparison between Post- and Pre-Intervention of Vitamin D**				
Kotsa *et al.*, (2009) [68]	Greece	ESHRE/ASRM	Obese PCOS, (15)	TC; TG; HDL-C; LDL-C
Selimoglu *et al.*, (2010) [69]	Turkey	ESHRE/ASRM	PCOS, (11)	FG; FI; HOMA-IR; 25/(OH)D; TT; FT; SHBG; DHEAS
Wehr *et al.*, (2011) [70]	Austria	ESHRE/ASRM	PCOS, (52)	FG; FI; HOMA-IR; HOMA-β; TC; TG; HDL-C; LDL-C; FT; SHBG; TT; FAI; 25(OH)D
Ardabili *et al.*, 2012 [71]	Iran	ESHRE/ASRM	PCOS and vitamin D deficiency, (50)	FG; FI; HOMA-IR; HOMA-β; QUICKI
Bonakdaran *et al.*, (2012) [66]	Iran	ESHRE/ASRM	PCOS, (51)	FG; FI; HOMA-IR; TT; DHEAS; 25(OH)D
Pal *et al.*, (2012) [72]	USA	ESHRE/ASRM	Overweight PCOS, (12)	FG; FI; QUICKI; TT; SHBG; FAI; 25(OH)D
Rahimi-Ardabili *et al.*, (2013) [73]	Iran	ESHRE/ASRM	PCOS and vitamin D deficiency, (50)	TC; TG; HDL-C; LDL-C; 25(OH)D
Asemi *et al.* (2014) [74]	Iran	ESHRE/ASRM	Overweight or obese PCOS, (52)	25(OH)D; FG; FI; HOMA-IR; QUICKI; TG; TC; LDL-C; HDL-C
Raja-Khan *et al.*, (2014) [75]	USA	NIH	PCOS, (28)	FG; FI; QUICKI; HOMA-IR; TC; HDL-C; LDL-C; TG; TT; FT; 25(OH)D
Tehrani *et al.* (2014) [76]	Iran	NIH		PCOS, (40)	25(OH)D
**Post-Intervention of Vitamin D Compared to Post-Intervention of Placebo**				
Ardabili *et al.*, 2012[71]	Iran	ESHRE/ASRM	PCOS and vitamin D deficiency, (50)	FG; FI; HOMA-IR; HOMA-β; QUICKI
Bonakdaran *et al.*, (2012) [66]	Iran	ESHRE/ASRM	PCOS, (51)	FG; FI; HOMA-IR; TT; DHEAS; 25(OH)D
Rahimi-Ardabili *et al.*, (2013) [73]	Iran	ESHRE/ASRM	PCOS and vitamin D deficiency, (50)	TC; TG; HDL-C; LDL-C; 25(OH)D
Asemi *et al.* (2014) [74]	Iran	ESHRE/ASRM	Overweight or obese PCOS, (52)	25(OH)D; FG; FI; HOMA-IR; QUICKI; TG; LDL-C; HDL-C
Raja-Khan *et al.*, (2014) [75]	USA	NIH	PCOS, (28)	FG; FI; QUICKI; HOMA-IR; TC; HDL-C; LDL-C; TG; TT; FT; 25(OH)D
Tehrani *et al.* (2014) [76]	Iran	NIH	PCOS, (40)	25(OH)D

PCOS: polycystic ovary syndrome; CTRL: control; 25(OH)D: 25-hydroxyvitamin D; 1,25(OH)_2_D: 1,25-dihydroxyvitamin D; FG: fasting glucose; FI: fasting insulin; HOMA-IR: homeostatic model assessment-insulin resistance index; HOMA-β:homeostatic model assessment- β-cell functions ; QUICKI: quantitative insulin sensitivity check index; TC: total cholesterol; HDL-C: high density lipoprotein cholesterol; LDL-C: low density lipoprotein cholesterol; TG: triglycerides; TT: total testosterone; FT: free testosterone; CRP: C-reactive protein; FAI: free androgen index; SHBG: sex hormone-binding; DHEAS: dehydroepiandrosterone sulfate; ESHRE/ASRM: European Society for Human Reproduction & Embryology/American Society for Reproduction Medicine.

### 3.5. Correlation of 25(OH)D with Metabolic and/or Endocrine Parameters

As shown in Table 2, serum 25(OH)D concentrations were negatively correlated with fasting glucose, fasting insulin, triglycerides, CRP, FAI and DHEAS among women with PCOS. Positive correlations between 25(OH)D and QUICKI, HDL-C, and SHBG, were found. Significant heterogeneity was identified from parameters including fasting glucose, HOMA-IR, total cholesterol, HDL-C, LDL-C, triglycerides, total testosterone, free testosterone and DHEAS. To investigate unduly influential studies, a sensitivity analysis was performed on parameters that showed substantial between-study heterogeneity and had been reported by five or more included studies (data not shown). When excluding the study conducted by Yildizhan *et al.* [65], no significant changes were detected in either pooled effect size estimate (*r*: −0.26; 95% CI: −0.36 to −0.15) or between-study heterogeneity (*p* = 0.027, *I^2^* = 53.8%) over studies reporting HOMA-IR. Similarly, no substantially influential studies were identified among those reporting fasting glucose, total cholesterol, HDL-C, LDL-C and FAI in terms of results of sensitivity tests. For studies reporting triglycerides, omitting the study by Yildizhan *et al.* [65] led to substantial change in heterogeneity test (*p* = 0.325, *I^2^* = 14.0%), but not in the estimate of effect size (*r*: −0.20; 95%CI: −0.30 to −0.10). Likewise, a significant change was identified in the heterogeneity test for total testosterone by removing observations obtained by Yildizhan *et al.* [65]. Four studies reported correlation coefficients of 25(OH)D with biochemical parameters [53,57,58,60] among non-PCOS controls. We found negative correlations between 25(OH)D levels and fasting insulin, HOMA-IR, CRP. Additionally, QUICKI was found to be positively correlated with 25(OH)D levels (data not shown).

### 3.6. Comparison between Post- and Pre-Intervention of Vitamin D Supplementation

Triglyceride levels in PCOS patients were significantly decreased, and blood 25(OH)D levels were significantly increased after treatment with vitamin D (Table 2). Substantially statistical heterogeneity was found among included studies reporting 25(OH)D, but no extremely influential studies were identified in terms of sensitivity tests. No significant publication bias was found in all reported parameters except triglycerides.

### 3.7. Post-Intervention of Vitamin D Compared to Post-Intervention of Placebo

Fasting insulin levels in PCOS patients were significantly higher in vitamin D intervention group compared to placebo group (Table 2). No significant differences were found in other metabolic parameters and serum 25(OH)D levels between treatment and placebo groups. No significant publication bias was found among reported parameters.

**Table 2:** Meta-analysis results.

Outcome	No. of Studies	No. of Observations	SMD (95%CI) ^‡^	Heterogeneity Test		Publication Bias
				*p*-Value	*I*^2^ (%)	*p*-Value
**Comparison between Vitamin D Deficient and Non-Deficient PCOS Patients**						
Fasting glucose	4	5	0.31 (0.10, 0.53)	0.429	0.0	0.254
Fasting insulin	4	5	0.63 (0.42, 0.85)	0.146	41.3	0.077
HOMA-IR	4	5	1.11 (0.51, 1.71)	0.002	76.5	0.130
HOMA-β	2	3	0.43 (0.15, 0.71)	0.183	41.1	0.613
QUICKI	2	3	−0.63 (−1.28, 0.03)	0.069	62.6	0.207
Total cholesterol	3	4	−0.14 (−0.67, 0.40)	0.026	67.7	0.767
HDL-C	2	3	−0.58 (−0.86, −0.30)	0.379	0.0	0.673
LDL-C	2	3	−0.11 (−0.39, 0.16)	0.101	56.3	0.658
Triglycerides	3	4	−0.17 (−1.33, 0.99)	<0.001	92.7	0.657
CRP	3	4	0.12 (−0.67, 0.92)	<0.001	85.2	0.757
Total testosterone	4	4	0.08 (−0.28, 0.60)	0.075	56.5	0.576
SHBG	4	4	0.16 (−0.28, 0.60)	0.018	70.1	0.656
FAI	3	3	0.25 (0.01, 0.48)	0.385	0.0	0.281
**Correlation between 25(OH)D and Metabolic and Endocrine Parameters among Women with PCOS**						
Fasting glucose	6	6	−0.23 (−0.38, −0.07)	0.009	67.2	0.287
Fasting insulin	6	6	−0.29 (−0.37, −0.21)	0.274	21.2	0.410
HOMA-IR	9	10	−0.52 (−0.23, 0.72)	<0.001	95.9	0.153
HOMA-β	3	3	−0.01 (−0.13, 0.11)	0.351	4.4	0.335
QUICKI	3	3	0.19 (0.07, 0.30)	0.467	0.0	0.036
Total cholesterol	5	6	−0.05 (−0.30, 0.21)	<0.001	84.4	0.812
HDL-C	5	5	0.35 (0.22, 0.47)	0.079	52.2	0.955
LDL-C	5	5	−0.06 (−0.33, 0.21)	<0.001	86.4	0.762
Triglycerides	6	7	−0.69 (−0.91, −0.16)	<0.001	98.4	0.272
CRP	3	3	−0.28 (−0.37, −0.18)	0.550	0.0	0.530
Total testosterone	5	6	−0.65 (−0.94, 0.18)	<0.001	98.8	0.422
SHBG	5	5	0.31 (0.23, 0.39)	0.179	36.4	0.815
FAI	5	5	−0.22 (−0.31, −0.12)	0.099	48.8	0.406
Free testosterone	2	2	−0.14 (−0.41, −0.15)	0.007	86.1	NA
DHEAS	5	6	−0.68 (−0.90, −0.17)	<0.001	97.6	0.259
**Comparison between Post- and Pre-Intervention of Vitamin D**						
Fasting glucose	7	7	−0.14 (−0.37, 0.09)	0.285	19.0	0.101
Fasting insulin	7	7	−0.02 (−0.25, 0.21)	0.838	0.0	0.722
HOMA-IR	6	6	−0.05 (−0.29, 0.19)	0.692	0.0	0.644
HOMA-β	3	3	0.16 (−0.12, 0.44)	0.767	0.0	0.953
QUICKI	4	4	−0.07 (−0.39, 0.26)	0.762	0.0	0.579
Total cholesterol	5	5	0.01 (−0.24, 0.26)	0.374	5.7	0.643
HDL-C	5	5	0.03 (−0.22, 0.27)	0.768	0.0	0.174
LDL-C	4	4	0.10 (−0.17, 0.38)	0.312	0.0	0.577
Triglycerides	4	4	−0.45 (−0.73, −0.17)	0.607	0.0	0.002
Total testosterone	5	5	−0.07 (−0.35, 0.21)	0.844	0.0	0.732
SHBG	3	3	−0.16 (−0.49, 0.17)	0.729	0.0	0.706
FAI	2	2	−0.14 (−0.49, 0.22)	0.429	0.0	NA
Free testosterone	3	3	−0.17 (−0.50, 0.16)	0.416	0.0	0.649
DHEAS	2	2	0.16 (−0.39, 0.70)	0.492	0.0	NA
25(OH)D	7	7	2.09 (1.28, 2.91)	<0.001	85.5	0.201
**Post-Intervention of Vitamin D Compared to Post-Intervention of Placebo**						
Fasting glucose	4	4	0.27 (−0.04, 0.58)	0.210	33.7	0.811
Fasting insulin	4	4	0.14 (−0.14, 0.45)	0.116	49.3	0.513
HOMA-IR	4	4	0.25 (−0.07, 0.56)	0.760	0.0	0.440
QUICKI	3	3	−0.14 (−0.48, 0.21)	0.710	0.0	0.254
HDL-C	3	4	0.22 (−0.22, 0.66)	0.095	52.9	0.392
LDL-C	3	3	−0.11 (0.46, 0.23)	0.799	0.0	0.133
Triglycerides	3	3	−0.04 (−0.38, 0.31)	0.474	0.0	0.474
Total testosterone	2	2	−0.09 (−0.60, 0.42)	0.404	0.0	NA
25(OH)D	5	5	2.11 (0.85, 3.37)	<0.001	79.5	0.177

25(OH)D: 25-hydroxyvitamin D; HOMA-IR: homeostatic model assessment-insulin resistance index; HOMA-β: homeostatic model assessment- β-cell functions ; QUICKI: quantitative insulin sensitivity check index; TC: total cholesterol; HDL-C: high density lipoprotein cholesterol; LDL-C: low density lipoprotein cholesterol; CRP: C-reactive protein; FAI: free androgen index; SHBG: sex hormone-binding; DHEAS: dehydroepiandrosterone sulfate. For comparisons between vitamin D deficient and non-deficient groups, SMD < 0 suggests a negative association with vitamin D deficiency, SMD > 0 indicates a positive association with vitamin D deficiency. For correlations of 25(OH)D with metabolic and endocrine parameters among women with PCOS, SMD < 0 suggests a negative correlation with 25(OH)D; SMD > 0 indicates a positive correlation with 25(OH)D. For comparisons between post- and pre-intervention of vitamin D, SMD < 0 suggests a negative effect of vitamin D supplementation, SMD>0 indicates a positive effect of vitamin D supplementation. For comparisons between post-invention of vitamin D and placebo groups, SMD < 0 suggests a negative effect of vitamin D supplementation, SMD > 0 indicates a positive effect of vitamin D supplementation.

## 4. Discussion

### 4.1. Principal Findings and Interpretations

The present review and meta-analysis supports that serum vitamin D status is related to metabolic and hormonal dysfunctions in women with PCOS. Among women with PCOS, those with vitamin D deficiency were more likely to have measures of dysglycemia compared to those without vitamin D deficiency. Similarly, lower vitamin D levels were positively correlated with markers of dysglycemia, support a significant difference in serum vitamin D levels between women with and without PCOS. In addition, we found no significant improvement in metabolic and hormonal functions among women with PCOS supplemented with vitamin D.

Metabolic disturbance is not uncommon in women affected by PCOS, with a prevalence of insulin resistance ranging from 50% to 70% [77,78,79], and up to 70% for dyslipidemia [10]. Dysglycemia and dyslipidemia, regular types of nutritional and metabolic disorders, are associated with elevated prevalence of insulin resistance, impaired glucose tolerance, metabolic syndrome, type 2 diabetes, and cardiovascular disease. A meta-analysis conducted by Moran *et al.* demonstrated that PCOS is associated with a higher prevalence of impaired glucose tolerance (OR: 2.48; 95% CI: 1.63 to 3.77), type 2 diabetes mellitus (OR: 4.43; 95% CI: 4.06 to 4.82), and metabolic syndrome (OR: 2.88; 95% CI: 2.40 to 3.45) [80]. Another meta-analysis revealed that the risk of cardiovascular disease was higher among women with PCOS compared to non-PCOS controls (RR:2.02; 95%CI: 1.47 to 2.76) [17]. Recently, the potential causes of metabolic dysregulation in PCOS have been the subject of intense scholarly debate. Serum vitamin D status has been proposed as a missing link between metabolic dysregulation and PCOS. The role of vitamin D on PCOS and its possible implication for metabolic dysfunctions among women with PCOS has been extensively studied in recent years. A body of evidence suggests that a low vitamin D level is related to elevated levels of HOMA-IR, total cholesterol, LDL-C, glucose, CRP, triglycerides, and decreased HDL-C, QUICKI in women affected by PCOS [22,48,52,53,57,58,65]. In agreement with these findings, the present study reports inverse associations of serum vitamin D concentrations with HOMA-IR, CRP, triglycerides, and positive associations with HDL-C, QUICKI.

Hyperandrogenism, one of the primary symptoms of PCOS, is characterized by excessive levels of androgens in the body. It is reported that approximately 75% of PCOS patients have hyperandrogenism, and more than 80% of which demonstrate supranormal levels of free testosterone [81]. Serum DHEAS, testosterone, SHBG and FAI are used as diagnostic markers of hyperandrogenism. There is evidence suggesting that low serum vitamin D levels are associated with abnormalities in markers of hyperandrogenism. For instance, some studies report inverse associations between serum 25(OH)D levels and testosterone, DHEAS and FAI and SHBG among with PCOS [22,53,63,64,65,72]. Consistent results were obtained from the present study.

VDD is common among women with PCOS [16], but the present meta-analysis does not demonstrate significantly lower levels of vitamin D among women with PCOS compared to non-PCOS controls. This finding, however, should be interpreted with caution. Through a close examination of included studies in this review, we find substantially varied prevalence of VDD among women with PCOS. A recent observational study by Velija-Asimi *et al.* found that 68% (41 out of 60) PCOS patients had VDD, of which 54% (*n* = 22) were obese and 46% were non-obese (*n* = 19) [63]. Another study also reported a higher prevalence of VDD in obese women with PCOS than in lean women with PCOS (70% *vs.* 60%) [56]. These studies suggest that the risk of VDD is associated with comorbidities among women with PCOS. That is, incidence of VDD is dependent upon severity of PCOS. Therefore, assessment of prevalence of VDD regardless of development stage of PCOS may be responsible for the insignificant difference in vitamin D status between PCOS and non-PCOS groups.

Epidemiologic studies suggest that low vitamin D levels are related to impaired glucose clearance, insulin secretion, and insulin resistance [82,83,84,85,86,87]. It is known that vitamin D affects glucose metabolism and may play a role in the development of subsequent metabolic and endocrine disorders in women with PCOS. Our study identified eight studies that investigated the effects of vitamin D therapy on metabolic and/or endocrine parameters on women with PCOS [66,68,69,70,71,72,73,75]. The results of this study suggest that supplementation of vitamin D does not significantly improve metabolic (except triglycerides) and endocrine features in PCOS patients. Similarly, no significant differences in metabolic parameters (except fasting insulin) were found between vitamin D supplementation and placebo groups. If a causal relationship exists, intervention of vitamin D is supposed to result in mitigation of metabolic and hormonal features in PCOS. In this regard, it is questionable that there is a cause-effect relationship between VDD and PCOS.

### 4.2. Strengths and Limitations

This study is the first meta-analysis summarizing evidence of the roles of vitamin D on metabolic and hormonal features in women with PCOS. The primary strength of this meta-analysis is the extensive literature search. Relevant studies from five popular databases were identified using a comprehensive search strategy. An additional strength is that sensitivity tests were implemented to identify influential studies, which is important because inclusion of influential studies can lead to inaccurate conclusions.

We also acknowledge the limitations of this literature review and meta-analysis. Despite a thorough search strategy, unavailable studies may exist which have not been included in the review. Because of a limited number of studies, it was not possible to assess publication bias over all metabolic and endocrine parameters, which limits our ability to perform subgroup analyses and attenuates the power of analyses. Included vitamin D intervention studies are subject to several limitations. On the one hand, most of the vitamin D supplementation studies were not randomized designs and were conducted with relatively small sizes of subjects, which may jeopardize the statistical power and reliability of corresponding findings. On the other hand, dosage of vitamin D supplementation and patients’ baseline serum vitamin D levels varied from study to study, thereby, there is uncertainty that all patients were administered sufficient amount of vitamin D. Moreover, seasonal and latitudinal changes play crucial impacts on cutaneous synthesis of vitamin D because solar ultraviolet B (UVB) radiation is one of major sources of vitamin D for humans. However, selected intervention studies failed to adjust for the confounding effects caused by seasonal and latitudinal disparities. Therefore, caution should be applied in extrapolation of results from the intervention studies. A further limitation of the present study is the lack of standardized scales for assessment of the quality of included studies, therefore the summarized effect size estimates should be interpreted with caution. Statistically significant heterogeneity was found from the majority of analyses (approximately 80%) concerning correlation between 25(OH)D levels and biochemical parameters, which may result from unreported findings (e.g., some studies reported only significant correlations) and/or unpublished literature. Finally, included studies varied in the definitions of VDD, which may impact the assessment of the role of VDD on metabolic and endocrine disorders in PCOS. For example, Patra *et al.* [61] and Velija-Asimi *et al.* [63] used 30 ng ml^−^^1^ and used 20 ng ml^−^^1^ as the cutoff points for vitamin D deficiency, respectively.

## 5. Conclusions

VDD is common among women affected by PCOS. If VDD were causally related to PCOS and the subsequent development of metabolic and hormonal dysfunction in PCOS, vitamin D supplementation would be a promising alternative for the prevention and treatment of PCOS. Meta-analysis of cross-sectional/case-control studies supports the existence of positive associations between VDD and metabolic and endocrine disorders in PCOS. Prospective observational studies that investigate the temporal relationship between VDD and PCOS are lacking. Our meta-analysis of intervention studies does not suggest a beneficial effect of vitamin D supplementation on metabolic and endocrine functions in women with PCOS despite the association noted in cross-sectional and case-control studies. Our review and meta-analysis therefore suggests that there is limited to no evidence that VDD is causally linked to development of PCOS. In light of this, we conclude that dysregulation of vitamin D metabolism may be a consequence of PCOS. Alternatively, VDD may be a common comorbid manifestation of PCOS. However, we cannot rule out the possibility that VDD may be a minor pathway towards PCOS given the small size of intervention studies and the variability of results from them. This review highlights a need for larger prospective studies in a well characterized sample of women with and without PCOS. Future studies should include multiple assessments of vitamin D in women at various stages of PCOS (if possible) to enhance our understanding of the temporal order of VDD in relation to PCOS, *i.e.*, is VDD as a determinant of PCOS metabolic and hormonal dysregulations, a consequence of PCOS metabolic and hormonal dysregulations, or both?

## Supplementary Files

Supplementary File 1

## References

[1] Rotterdam ESHRE/ASRM-Sponsored PCOS Consensus Workshop Group (2004). Revised 2003 consensus on diagnostic criteria and long-term health risks related to polycystic ovary syndrome (pcos). Hum. Reprod..

[2] Asuncion M., Calvo R.M., San Millan J.L., Sancho J., Avila S., Escobar-Morreale H.F. (2000). A prospective study of the prevalence of the polycystic ovary syndrome in unselected caucasian women from spain. J. Clin. Endocrinol. Metab..

[3] Diamanti-Kandarakis E., Kouli C.R., Bergiele A.T., Filandra F.A., Tsianateli T.C., Spina G.G., Zapanti E.D., Bartzis M.I. (1999). A survey of the polycystic ovary syndrome in the greek island of lesbos: Hormonal and metabolic profile. J. Clin. Endocrinol. Metab..

[4] Azziz R., Woods K.S., Reyna R., Key T.J., Knochenhauer E.S., Yildiz B.O. (2004). The prevalence and features of the polycystic ovary syndrome in an unselected population. J. Clin. Endocrinol. Metab..

[5] March W.A., Moore V.M., Willson K.J., Phillips D.I., Norman R.J., Davies M.J. (2010). The prevalence of polycystic ovary syndrome in a community sample assessed under contrasting diagnostic criteria. Hum. Reprod..

[6] Grulet H., Hecart A.C., Delemer B., Gross A., Sulmont V., Leutenegger M., Caron J. (1993). Roles of lh and insulin resistance in lean and obese polycystic ovary syndrome. Clin. Endocrinol..

[7] Diamanti-Kandarakis E., Dunaif A. (1996). New perspectives in polycystic ovary syndrome. Trends Endocrinol. Metab..

[8] Dunaif A., Finegood D.T. (1996). Beta-cell dysfunction independent of obesity and glucose intolerance in the polycystic ovary syndrome. J. Clin. Endocrinol. Metab..

[9] Alemzadeh R., Kansra A.R. (2011). New adolescent polycystic ovary syndrome perspectives. Minerva Pediatr..

[10] Legro R.S., Kunselman A.R., Dunaif A. (2001). Prevalence and predictors of dyslipidemia in women with polycystic ovary syndrome. Am. J. Med..

[11] Morin-Papunen L.C., Vauhkonen I., Koivunen R.M., Ruokonen A., Tapanainen J.S. (2000). Insulin sensitivity, insulin secretion, and metabolic and hormonal parameters in healthy women and women with polycystic ovarian syndrome. Hum. Reprod..

[12] Wild R.A., Rizzo M., Clifton S., Carmina E. (2011). Lipid levels in polycystic ovary syndrome: Systematic review and meta-analysis. Fertil. Steril..

[13] Forrest K.Y., Stuhldreher W.L. (2011). Prevalence and correlates of vitamin d deficiency in us adults. Nutr. Res..

[14] Hovsepian S., Amini M., Aminorroaya A., Amini P., Iraj B. (2011). Prevalence of vitamin d deficiency among adult population of Isfahan city, Iran. J. Health Popul. Nutr..

[15] Tangpricha V., Pearce E.N., Chen T.C., Holick M.F. (2002). Vitamin d insufficiency among free-living healthy young adults. Am. J. Med..

[16] Thomson R.L., Spedding S., Buckley J.D. (2012). Vitamin D in the aetiology and management of polycystic ovary syndrome. Clin. Endocrinol..

[17] De Groot P.C., Dekkers O.M., Romijn J.A., Dieben S.W., Helmerhorst F.M. (2011). Pcos, coronary heart disease, stroke and the influence of obesity: A systematic review and meta-analysis. Hum. Reprod. Update.

[18] Khan H., Kunutsor S., Franco O.H., Chowdhury R. (2013). Vitamin D, type 2 diabetes and other metabolic outcomes: A systematic review and meta-analysis of prospective studies. Proc. Nutr. Soc..

[19] Song Y., Wang L., Pittas A.G., Del Gobbo L.C., Zhang C., Manson J.E., Hu F.B. (2013). Blood 25-hydroxy vitamin d levels and incident type 2 diabetes: A meta-analysis of prospective studies. Diabetes Care.

[20] Verdoia M., Schaffer A., Sartori C., Barbieri L., Cassetti E., Marino P., Galasso G., De Luca G. (2014). Vitamin D deficiency is independently associated with the extent of coronary artery disease. Eur. J. Clin. Investig..

[21] Krul-Poel Y.H., Snackey C., Louwers Y., Lips P., Lambalk C.B., Laven J.S., Simsek S. (2013). The role of vitamin D in metabolic disturbances in polycystic ovary syndrome: A systematic review. Eur. J. Endocrinol..

[22] Wehr E., Pilz S., Schweighofer N., Giuliani A., Kopera D., Pieber T.R., Obermayer-Pietsch B. (2009). Association of hypovitaminosis D with metabolic disturbances in polycystic ovary syndrome. Eur. J. Endocrinol..

[23] Holick M.F. (2007). Vitamin D deficiency. N. Engl. J. Med..

[24] Ramagopalan S.V., Heger A., Berlanga A.J., Maugeri N.J., Lincoln M.R., Burrell A., Handunnetthi L., Handel A.E., Disanto G., Orton S.M. (2010). A chip-seq defined genome-wide map of vitamin d receptor binding: Associations with disease and evolution. Genome Res..

[25] Dobnig H., Pilz S., Scharnagl H., Renner W., Seelhorst U., Wellnitz B., Kinkeldei J., Boehm B.O., Weihrauch G., Maerz W. (2008). Independent association of low serum 25-hydroxyvitamin D and 1,25-dihydroxyvitamin D levels with all-cause and cardiovascular mortality. Arch. Intern. Med..

[26] Freedman D.M., Looker A.C., Chang S.C., Graubard B.I. (2007). Prospective study of serum vitamin D and cancer mortality in the united states. J. Natl. Cancer Inst..

[27] Zittermann A., Schleithoff S.S., Tenderich G., Berthold H.K., Korfer R., Stehle P. (2003). Low vitamin D status: A contributing factor in the pathogenesis of congestive heart failure?. J. Am. Coll. Cardiol..

[28] Teegarden D., Donkin S.S. (2009). Vitamin D: Emerging new roles in insulin sensitivity. Nutr. Res. Rev..

[29] Plymate S.R., Matej L.A., Jones R.E., Friedl K.E. (1988). Inhibition of sex hormone-binding globulin production in the human hepatoma (Hep G2) cell line by insulin and prolactin. J. Clin. Endocrinol. Metabol..

[30] Haffner S.M., Stern M.P., Mitchell B.D., Hazuda H.P., Patterson J.K. (1990). Incidence of type II diabetes in mexican americans predicted by fasting insulin and glucose levels, obesity, and body-fat distribution. Diabetes.

[31] Lehto S., Ronnemaa T., Pyorala K., Laakso M. (2000). Cardiovascular risk factors clustering with endogenous hyperinsulinaemia predict death from coronary heart disease in patients with type II diabetes. Diabetologia.

[32] Lillioja S., Mott D.M., Spraul M., Ferraro R., Foley J.E., Ravussin E., Knowler W.C., Bennett P.H., Bogardus C. (1993). Insulin resistance and insulin secretory dysfunction as precursors of non-insulin-dependent diabetes mellitus. Prospective studies of pima indians. N. Engl. J. Med..

[33] Ruige J.B., Assendelft W.J., Dekker J.M., Kostense P.J., Heine R.J., Bouter L.M. (1998). Insulin and risk of cardiovascular disease: A meta-analysis. Circulation.

[34] Rutter M.K., Meigs J.B., Sullivan L.M., D’Agostino R.B., Wilson P.W. (2005). Insulin resistance, the metabolic syndrome, and incident cardiovascular events in the framingham offspring study. Diabetes.

[35] Moher D., Liberati A., Tetzlaff J., Altman D.G., Group P. (2009). Preferred reporting items for systematic reviews and meta-analyses: The prisma statement. PLoS Med..

[36] Stroup D.F., Berlin J.A., Morton S.C., Olkin I., Williamson G.D., Rennie D., Moher D., Becker B.J., Sipe T.A., Thacker S.B. (2000). Meta-analysis of observational studies in epidemiology: A proposal for reporting. Meta-analysis of observational studies in epidemiology (moose) group. JAMA.

[37] Hartling L., Milne A., Hamm M.P., Vandermeer B., Ansari M., Tsertsvadze A., Dryden D.M. (2013). Testing the newcastle ottawa scale showed low reliability between individual reviewers. J. Clin. Epidemiol..

[38] Juni P., Altman D.G., Egger M. (2001). Systematic reviews in health care: Assessing the quality of controlled clinical trials. BMJ.

[39] Lo C.K., Mertz D., Loeb M. (2014). Newcastle-ottawa scale: Comparing reviewers’ to authors’ assessments. BMC Med. Res. Methodol..

[40] Cohen J. (1988). Statistical Power Analysis of the Behavioural Sciences.

[41] Panidis D., Balaris C., Farmakiotis D., Rousso D., Kourtis A., Balaris V., Katsikis I., Zournatzi V., Diamanti-Kandarakis E. (2005). Serum parathyroid hormone concentrations are increased in women with polycystic ovary syndrome. Clin. Chem..

[42] Hedges L.V., Olkin I. (1985). Statistical Methods for Meta-Analysis.

[43] Hozo S.P., Djulbegovic B., Hozo I. (2005). Estimating the mean and variance from the median, range, and the size of a sample. BMC Med. Res. Methodol..

[44] Rupinski M.T., Dunlap W.P. (1996). Approximating pearson product-moment correlations from kendall’s tau and spearman’s rho. Educ. Psychol. Meas..

[45] Higgins J.P., Thompson S.G., Deeks J.J., Altman D.G. (2003). Measuring inconsistency in meta-analyses. BMJ.

[46] DerSimonian R., Laird N. (1986). Meta-analysis in clinical trials. Control. Clin. Trials.

[47] Egger M., Davey Smith G., Schneider M., Minder C. (1997). Bias in meta-analysis detected by a simple, graphical test. BMJ.

[48] Hassan N.E., El-Orabi H.A., Eid Y.M., Mohammed N.R. (2012). Effect of 25-hydroxyvitamin D on metabolic parameters and insulin resistance in patients with polycystic ovarian syndrome. Middle East Fertil. Soc. J..

[49] Zawadski J.K., Dunaif A., Givens J.H.F., Merriman G. (1992). In The Polycystic Ovary Syndrome.

[50] Savastano S., Valentino R., di Somma C., Orio F., Pivonello C., Passaretti F., Brancato V., Formisano P., Colao A., Beguinot F. (2011). Serum 25-hydroxyvitamin D levels, phosphoprotein enriched in diabetes gene product (ped/pea-15) and leptin-to-adiponectin ratio in women with pcos. Nutr. Metab..

[51] Nestler J.E., Reilly E.R., Cheang K.I., Bachmann L.M., Downs R.W. (2012). A pilot study: Effects of decreasing serum insulin with diazoxide on vitamin D levels in obese women with polycystic ovary syndrome. Trans. Am. Clin. Climatol. Assoc..

[52] Mahmoudi T., Gourabi H., Ashrafi M., Yazdi R.S., Ezabadi Z. (2010). Calciotropic hormones, insulin resistance, and the polycystic ovary syndrome. Fertil. Steril..

[53] Li H.W., Brereton R.E., Anderson R.A., Wallace A.M., Ho C.K. (2011). Vitamin D deficiency is common and associated with metabolic risk factors in patients with polycystic ovary syndrome. Metab. Clin. Exp..

[54] Lin M.W., Tsai S.J., Chou P.Y., Huang M.F., Sun H.S., Wu M.H. (2012). Vitamin D receptor 1a promotor −1521 g/c and −1012 a/g polymorphisms in polycystic ovary syndrome. Taiwan J. Obstet. Gynecol..

[55] Mazloomi S., Sharifi F., Hajihosseini R., Kalantari S., Mazloomzadeh S. (2012). Association between hypoadiponectinemia and low serum concentrations of calcium and vitamin D in women with polycystic ovary syndrome. ISRN Endocrinol..

[56] Tsakova A.D., Gateva A.T., Kamenov Z.A. (2012). 25(OH) vitamin D levels in premenopausal women with polycystic ovary syndrome and/or obesity. Int. J. Vitam. Nutr. Res..

[57] El-Shal A.S., Shalaby S.M., Aly N.M., Rashad N.M., Abdelaziz A.M. (2013). Genetic variation in the vitamin D receptor gene and vitamin D serum levels in egyptian women with polycystic ovary syndrome. Mol. Biol. Rep..

[58] Guducu N., Gormus U., Kutay S.S., Kavak Z.N., Dunder I. (2014). 25-hydroxyvitamin D levels are related to hyperinsulinemia in polycystic ovary syndrome. Gynecol. Endocrinol..

[59] Ghadimi R., Esmaeilzadeh S., Firoozpour M., Ahmadi A. (2014). Does vitamin D status correlate with clinical and biochemical features of polycystic ovarysyndrome in high school girls?. Casp. J. Intern. Med..

[60] Sahin S., Eroglu M., Selcuk S., Turkgeldi L., Kozali S., Davutoglu S., Muhcu M. (2014). Intrinsic factors rather than vitamin D deficiency are related to insulin resistance in lean women with polycystic ovary syndrome. Eur. Rev. Med. Pharmacol. Sci..

[61] Patra S.K., Nasrat H., Goswami B., Jain A. (2012). Vitamin D as a predictor of insulin resistance in polycystic ovarian syndrome. Diabetes Metab. Syndr..

[62] Bhattacharya S.M., Jha A. (2013). Association of vitamin D3 deficiency with clinical and biochemical parameters in indian women with polycystic ovary syndrome. Int. J. Gynaecol. Obstet..

[63] Velija-Asimi Z. (2014). Evaluation of the association of vitamin D deficiency with gonadotropins and sex hormone in obese and non-obese women with polycystic ovary syndrome. Med. Glas..

[64] Hahn S., Haselhorst U., Tan S., Quadbeck B., Schmidt M., Roesler S., Kimmig R., Mann K., Janssen O.E. (2006). Low serum 25-hydroxyvitamin D concentrations are associated with insulin resistance and obesity in women with polycystic ovary syndrome. Exp. Clin. Endocrinol. Diabetes.

[65] Yildizhan R., Kurdoglu M., Adali E., Kolusari A., Yildizhan B., Sahin H.G., Kamaci M. (2009). Serum 25-hydroxyvitamin D concentrations in obese and non-obese women with polycystic ovary syndrome. Arch. Gynecol. Obstet..

[66] Bonakdaran S., Khorasani Z.M., Davachi B., Khorasani J.M. (2012). The effects of calcitriol on improvement of insulin resistance, ovulation and comparison with metformin therapy in pcos patients: A randomized placebo-controlled clinical trial. Iran. J. Reprod. Med..

[67] Kozakowski J., Kapuscinska R., Zgliczynski W. (2014). Associations of vitamin D concentration with metabolic and hormonal indices in women with polycystic ovary syndrome presenting abdominal and gynoidal type of obesity. Ginekol. Pol..

[68] Kotsa K., Yavropoulou M.P., Anastasiou O., Yovos J.G. (2009). Role of vitamin D treatment in glucose metabolism in polycystic ovary syndrome. Fertil. Steril..

[69] Selimoglu H., Duran C., Kiyici S., Ersoy C., Guclu M., Ozkaya G., Tuncel E., Erturk E., Imamoglu S. (2010). The effect of vitamin D replacement therapy on insulin resistance and androgen levels in women with polycystic ovary syndrome. J. Endocrinol. Investig..

[70] Wehr E., Pieber T.R., Obermayer-Pietsch B. (2011). Effect of vitamin D3 treatment on glucose metabolism and menstrual frequency in polycystic ovary syndrome women: A pilot study. J. Endocrinol. Investig..

[71] Ardabili H.R., Gargari B.P., Farzadi L. (2012). Vitamin D supplementation has no effect on insulin resistance assessment in women with polycystic ovary syndrome and vitamin D deficiency. Nutr. Res..

[72] Pal L., Berry A., Coraluzzi L., Kustan E., Danton C., Shaw J., Taylor H. (2012). Therapeutic implications of vitamin D and calcium in overweight women with polycystic ovary syndrome. Gynecol. Endocrinol..

[73] Rahimi-Ardabili H., Pourghassem Gargari B., Farzadi L. (2013). Effects of vitamin D on cardiovascular disease risk factors in polycystic ovary syndrome women with vitamin D deficiency. J. Endocrinol. Investig..

[74] Asemi Z., Foroozanfard F., Hashemi T., Bahmani F., Jamilian M., Esmaillzadeh A. (2014). Calcium plus vitamin D supplementation affects glucose metabolism and lipid concentrations in overweight and obese vitamin D deficient women with polycystic ovary syndrome. Clin. Nutr..

[75] Raja-Khan N., Shah J., Stetter C.M., Lott M.E., Kunselman A.R., Dodson W.C., Legro R.S. (2014). High-dose vitamin D supplementation and measures of insulin sensitivity in polycystic ovary syndrome: A randomized, controlled pilot trial. Fertil. Steril..

[76] Tehrani H.G., Mostajeran F., Shahsavari S. (2014). The effect of calcium and vitamin D supplementation on menstrual cycle, body mass index and hyperandrogenism state of women with poly cystic ovarian syndrome. J. Res. Med. Sci..

[77] Carmina E., Lobo R.A. (2004). Use of fasting blood to assess the prevalence of insulin resistance in women with polycystic ovary syndrome. Fertil. Steril..

[78] Dunaif A. (1997). Insulin resistance and the polycystic ovary syndrome: Mechanism and implications for pathogenesis. Endocr. Rev..

[79] Schachter M., Raziel A., Friedler S., Strassburger D., Bern O., Ron-El R. (2003). Insulin resistance in patients with polycystic ovary syndrome is associated with elevated plasma homocysteine. Hum. Reprod..

[80] Moran L.J., Misso M.L., Wild R.A., Norman R.J. (2010). Impaired glucose tolerance, type 2 diabetes and metabolic syndrome in polycystic ovary syndrome: A systematic review and meta-analysis. Hum. Reprod. Update.

[81] Huang A., Brennan K., Azziz R. (2010). Prevalence of hyperandrogenemia in the polycystic ovary syndrome diagnosed by the national institutes of health 1990 criteria. Fertil. Steril..

[82] Bourlon P.M., Billaudel B., Faure-Dussert A. (1999). Influence of vitamin D3 deficiency and 1,25 dihydroxyvitamin D3 on *de novo* insulin biosynthesis in the islets of the rat endocrine pancreas. J. Endocrinol..

[83] Liu E., Meigs J.B., Pittas A.G., McKeown N.M., Economos C.D., Booth S.L., Jacques P.F. (2009). Plasma 25-hydroxyvitamin D is associated with markers of the insulin resistant phenotype in nondiabetic adults. J. Nutr..

[84] Mattila C., Knekt P., Mannisto S., Rissanen H., Laaksonen M.A., Montonen J., Reunanen A. (2007). Serum 25-hydroxyvitamin D concentration and subsequent risk of type 2 diabetes. Diabetes Care.

[85] Ortlepp J.R., Metrikat J., Albrecht M., von Korff A., Hanrath P., Hoffmann R. (2003). The vitamin D receptor gene variant and physical activity predicts fasting glucose levels in healthy young men. Diabetic Med..

[86] Scragg R., Sowers M., Bell C., Third National H., Nutrition Examination S. (2004). Serum 25-hydroxyvitamin D, diabetes, and ethnicity in the third national health and nutrition examination survey. Diabetes Care.

[87] Zeitz U., Weber K., Soegiarto D.W., Wolf E., Balling R., Erben R.G. (2003). Impaired insulin secretory capacity in mice lacking a functional vitamin D receptor. FASEB J..

