# Supplementary material for: Serum Vitamin D Levels and Polycystic Ovary syndrome: A Systematic Review and Meta-Analysis
Source: Nutrients. 2015 Jun 8;7(6):4555–77. doi: 10.3390/nu7064555 (PMC4488802; doi:10.3390/nu7064555)
Supplement: Supplementary File 1 [file nutrients-07-04555-s001.docx]

**Supplementary Information**

**Table S1.** Database literature search.

|  | **Search Strings** | **Hits** |
| --- | --- | --- |
| PubMed | | |
| #1 | Polycystic ovary syndrome [MeSH Terms] | 10,411 |
| #2 | Polycystic ovary syndrome [tw] | 12,176 |
| #3 | polycystic ovarian syndrome [tw] | 1,696 |
| #4 | Polycystic ovary disease [tw] | 166 |
| #5 | polycystic ovarian disease [tw] | 617 |
| #6 | polycystic ovarian [tw] | 2,452 |
| #7 | Stein Leventhal Syndrome [tw] | 737 |
| #8 | Sclerocystic Ovarian Degeneration [tw] | 2 |
| #9 | Ovarian Degeneration [tw] | 38 |
| #10 | Sclerocystic Ovary [tw] | 41 |
| #11 | #1 OR #2 OR #3 OR #4 OR #5 OR #6 OR #7 OR #8 OR #9 OR #10 | 13,027 |
| #12 | pco [tw] | 3,989 |
| #13 | pcos [tw] | 6,708 |
| #14 | pcod [tw] | 265 |
| #15 | #12 OR #13 OR #14 | 10,622 |
| #16 | ovary [tw] | 107,400 |
| #17 | ovarian [tw] | 153,557 |
| #18 | ovaries [tw] | 28,344 |
| #19 | ovarial [tw] | 334 |
| #20 | #16 OR #27 OR #18 OR #19 | 221,643 |
| #21 | #15 AND #20 | 7,326 |
| #22 | #11 OR #21 | 13,152 |
| #23 | vitamin d [MeSH Terms] | 43,963 |
| #24 | cholecalciferol [MeSH Terms] | 22,478 |
| #25 | ergocalciferol [MeSH Terms] | 3,348 |
| #26 | #23 OR #24 OR #25 | 43,963 |
| #27 | vitamin d [tw] | 49,505 |
| #28 | cholecalciferol [tw] | 6,327 |
| #29 | ergocalciferol [tw] | 589 |
| #30 | alfacalcidol [tw] | 1,089 |
| #31 | alphacalcidol [tw] | 87 |
| #32 | 25 hydroxyvitamin D [tw] | 8,443 |
| #33 | vitamin D2 [tw] | 1,109 |
| #34 | vitamin D3 [tw] | 7,730 |
| #35 | 1-alpha hydroxyvitamin D3 [tw] | 282 |
| #36 | 25-hydroxycholecalciferol [tw] | 941 |
| #37 | 1,25 dihydroxyvitamin D3 [tw] | 4,548 |
| #38 | 1,25 dihydroxycholecalciferol [tw] | 1,100 |
| #39 | 25(OH)D [tw] | 13,335 |
| #40 | 1,25(OH)2D [tw] | 1,556 |

**Table S1.** *Cont.*

|  | **Search Strings** |  |
| --- | --- | --- |
| **PubMed** | | |
| #41 | vit D [tw] | 205 |
| #42 | 25OHD [tw] | 934 |
| #43 | #27 OR #28 OR #29 OR #30 #31 OR #32 OR #33 OR #34 OR #35 OR #36 OR #37 OR #38 OR #39 OR #40 OR #41 OR #42 | 60,571 |
| #44 | #26 OR #43 | 65,594 |
| #45 | #22 AND #44 | 101 |
| **Web of Science** | | |
| #1 | TOPIC: (Polycystic ovary syndrome) | 42,367 |
| #2 | TOPIC: (polycystic ovarian syndrome) | 17,419 |
| #3 | TOPIC: (Polycystic ovary disease) | 18,366 |
| #4 | TOPIC: (polycystic ovarian disease) | 9388 |
| #5 | TOPIC: (polycystic ovarian) | 20,254 |
| #6 | TOPIC: (Stein Leventhal Syndrome) | 1098 |
| #7 | TOPIC: (Sclerocystic Ovarian Degeneration) | 5 |
| #8 | TOPIC: (Ovarian Degeneration) | 3119 |
| #9 | TOPIC: (Sclerocystic Ovary) | 131 |
| #10 | #1 OR #2 OR #3 OR #4 OR #5 OR #6 OR #7 OR #8 OR #9 | 54,404 |
| #11 | TOPIC: (pco *) | 82,785 |
| #12 | TOPIC: (ovar *) | 969,951 |
| #13 | #11 AND #12 | 24,430 |
| #14 | #10 OR #13 | 55,078 |
| #15 | TOPIC: (vitamin d) | 250,433 |
| #16 | TOPIC: (vitamin d2) | 5294 |
| #17 | TOPIC: (vitamin d3) | 40,263 |
| #18 | TOPIC: (cholecalciferol) | 13,465 |
| #19 | TOPIC: (ergocalciferol) | 1,182 |
| #20 | TOPIC: (alfacalcidol) | 1,514 |
| #21 | TOPIC: (alphacalcidol) | 173 |
| #22 | TOPIC: (25 hydroxyvitamin D) | 37,045 |
| #23 | TOPIC: (1-alpha hydroxyvitamin D3) | 2,341 |
| #24 | TOPIC: (25-hydroxycholecalciferol) | 1587 |
| #25 | TOPIC: (1,25 dihydroxyvitamin D3) | 22,528 |
| #26 | TOPIC: (1,25 dihydroxycholecalciferol) | 2,638 |
| #27 | TOPIC: (25(OH)D) | 50,948 |
| #28 | TOPIC: (1,25(OH)2D) | 3232 |
| #29 | TOPIC: (25OHD) | 1166 |
| #30 | TOPIC: (vit D) | 2491 |
| #31 | #15 OR #16 OR #17 OR #18 OR #19 OR #20 OR #21 OR #22 OR #23 OR #24 OR #25 OR #26 OR #27 OR #28 OR #29 OR #30 | 290,894 |
| #32 | #14 AND #31 | 215 |

**Table S1.** *Cont.*

|  | **Search Strings** |  |
| --- | --- | --- |
| **Cochrane Central Register of Controlled Trials (CENTRAL)** | | |
| #1 | Search All Text: (“Polycystic ovary syndrome” or “polycystic ovarian  syndrome or “polycystic ovary” or “polycystic ovary diease” or  “polycystic ovarian disease” or “polycystic ovarian” or “stein leventhal  syndrome” or “ovarian degeneration” or “sclerocystic ovary”) | 1371 |
| #2 | Search All Text: (“pco*” and “ovar*”) | 978 |
| #3 | Enter terms for search #1 or #2 | 1402 |
| #4 | Search All Text: (“vitamin d” or “vitamin d2” or “vitamin d3” or  “cholecalciferol” or “alfacalcidol” or “alfacalcidol” or “alphacalcidol” or  “25 hydroxyvitamin d” or “1-alpha hydroxyvitamin d3” or “25-hydroxycholecalciferol” or “vit D” or “25(OH)D” or “1,25(OH)2D” or “25OHD”) | 6666 |
| #5 | Enter terms for search #3 and #4 | 25 |
| **Cumulative Index to Nursing and Allied Health (CINAHL)** | | |
| #1 | TX: (Polycystic ovary syndrome) | 1139 |
| #2 | TX: (polycystic ovarian syndrome) | 298 |
| #3 | TX: (Polycystic ovary disease) | 212 |
| #4 | TX: (polycystic ovarian disease) | 72 |
| #5 | TX: (polycystic ovarian) | 306 |
| #6 | TX: (Stein Leventhal Syndrome) | 8 |
| #7 | TX: (Sclerocystic Ovarian Degeneration) | 0 |
| #8 | TX: (Ovarian Degeneration) | 22 |
| #9 | TX: (Sclerocystic Ovary) | 0 |
| #10 | #1 OR #2 OR #3 OR #4 OR #5 OR #6 OR #7 OR #8 OR #9 | 1204 |
| #11 | TX: (pco*) | 1115 |
| #12 | TX: (ovar*) | 9918 |
| #13 | #11 AND #12 | 377 |
| #14 | #10 OR #13 | 1207 |
| #15 | TX: (vitamin d) | 10,108 |
| #16 | TX: (vitamin d2) | 82 |
| #17 | TX: (vitamin d3) | 422 |
| #18 | TX: (cholecalciferol) | 318 |
| #19 | TX: (ergocalciferol) | 36 |
| #20 | TX: (alfacalcidol) | 26 |
| #21 | TX: (alphacalcidol) | 7 |
| #22 | TX: (25 hydroxyvitamin D) | 1007 |
| #23 | TX: (1-alpha hydroxyvitamin D3) | 17 |
| #24 | TX: (25-hydroxycholecalciferol) | 17 |
| #25 | TX: (1,25 dihydroxyvitamin D3) | 68 |
| #26 | TX: (1,25 dihydroxycholecalciferol) | 8 |
| #27 | TX: (25(OH)D) | 721 |
| #28 | TX: (1,25(OH)2D) | 115 |
| #29 | TX: (25OHD) | 111 |
| #30 | TX: (vit D) | 23 |

**Table S1.** *Cont.*

|  | **Search Strings** |  |
| --- | --- | --- |
| **Cumulative Index to Nursing and Allied Health (CINAHL)** | | |
| #31 | #15 OR #16 OR #17 OR #18 OR #19 OR #20 OR #21 OR #22 OR #23 OR  #24 OR #25 OR #26 OR #27 OR #28 OR #29 OR #30 | 10,230 |
| #32 | #14 AND #31 | 23 |
| **PsycINFO** | | |
| #1 | TX: (Polycystic ovary syndrome) | 198 |
| #2 | TX: (polycystic ovarian syndrome) | 81 |
| #3 | TX: (Polycystic ovary disease) | 62 |
| #4 | TX: (polycystic ovarian disease) | 22 |
| #5 | TX: (polycystic ovarian) | 91 |
| #6 | TX: (Stein Leventhal Syndrome) | 6 |
| #7 | TX: (Sclerocystic Ovarian Degeneration) | 0 |
| #8 | TX: (Ovarian Degeneration) | 16 |
| #9 | TX: (Sclerocystic Ovary) | 1 |
| #10 | #1 OR #2 OR #3 OR #4 OR #5 OR #6 OR #7 OR #8 OR #9 | 286 |
| #11 | TX: (pco*) | 895 |
| #12 | TX: (ovar*) | 6475 |
| #13 | #11 AND #12 | 159 |
| #14 | #10 OR #13 | 290 |
| #15 | TX: (vitamin d) | 1753 |
| #16 | TX: (vitamin d2) | 30 |
| #17 | TX: (vitamin d3) | 170 |
| #18 | TX: (cholecalciferol) | 26 |
| #19 | TX: (ergocalciferol) | 8 |
| #20 | TX: (alfacalcidol) | 5 |
| #21 | TX: (alphacalcidol ) | 0 |
| #22 | TX: (25 hydroxyvitamin D) | 245 |
| #23 | TX: (1-alpha hydroxyvitamin D3) | 0 |
| #24 | TX: (25-hydroxycholecalciferol) | 1 |
| #25 | TX: (1,25 dihydroxyvitamin D3) | 25 |
| #26 | TX: (1,25 dihydroxycholecalciferol) | 1 |
| #27 | TX: (25(OH)D) | 502 |
| #28 | TX: (1,25(OH)2D) | 31 |
| #29 | TX: (25OHD) | 48 |
| #30 | TX: (vit D) | 20 |
| #31 | #15 OR #16 OR #17 OR #18 OR #19 OR #20 OR #21 OR #22 OR #23 OR #24 OR #25 OR #26 OR #27 OR #28 OR #29 OR #30 | 2069 |
| #32 | #14 AND #31 | 1 |

© 2015 by the authors; licensee MDPI, Basel, Switzerland. This article is an open access article distributed under the terms and conditions of the Creative Commons Attribution license (http://creativecommons.org/licenses/by/4.0/).
